# Deep learning for automated segmentation of brain edema in meningioma after radiosurgery

**DOI:** 10.1186/s12880-025-01660-x

**Published:** 2025-04-22

**Authors:** Huai-Che Yang, Tzu-Chiang Peng, Zhi-Hong Chen, Cheng-Chia Lee, Hsiu-Mei Wu, I-Chun Lai, Ching-Jen Chen, Syu-Jyun Peng

**Affiliations:** 1 School of Medicine, College of Medicine, National Yang Ming Chiao Tung University, Taipei, Taiwan; 2 Department of Neurosurgery, Neurological Institute, Taipei Veterans General Hospital, Taipei, Taiwan; 3 Department of Electrical Engineering, National Central University, Taoyuan, Taiwan; 4 Department of Radiology, Taipei Veterans General Hospital, Taipei, Taiwan; 5 Department of Heavy Particles & Radiation Oncology, Taipei Veterans General Hospital, Taipei, Taiwan; 6 University of Texas Health Science Center at Houston, Houston, TX USA; 7 In-Service Master Program in Artificial Intelligence in Medicine, College of Medicine, Taipei Medical University, No. 250, Wuxing St., Xinyi Dist, Taipei City, 110 Taiwan; 8 Clinical Big Data Research Center, Taipei Medical University Hospital, Taipei Medical University, Taipei, Taiwan

**Keywords:** Meningioma, Brain edema, Gamma knife radiosurgery, Deep learning, Brain segmentation

## Abstract

**Background:**

Although gamma Knife radiosurgery (GKRS) is commonly used to treat benign brain tumors, such as meningioma, irradiating the surrounding brain tissue can lead to perifocal edema within a few months after the procedure. Volumetric assessment of perifocal edema is crucial for therapy planning and monitoring. Post-radiosurgery changes in perifocal edema, appearing as hyper-dense areas in magnetic resonance T2-weighted (T2w) images, are clearly identifiable; however, physicians lack tools to segment and quantify the volume of these T2w hyper-dense areas. This has hindered not only the quantification of severity but also research on edema growth and case differentiation.

**Methods:**

In this study, we trained a Mask Region-based Convolutional Neural Network (Mask R-CNN) to replace manual pre-processing in designating regions of interest. We also applied transfer learning to the DeepMedic deep learning model to facilitate the automatic segmentation and quantification of brain edema regions in images. The resulting quantitative findings were used to explore the effects of GKRS treatment on brain edema caused by meningioma.

**Results:**

We studied 21 patients with meningiomas who had undergone GKRS treatment based on 154 regularly tracked T2w scans. From this group, we selected 130 scans for random assignment to a training set (80 scans), validation set (30 scans), and test set (20 scans). The actual range of the edema in the T2w images was labeled manually by a clinical radiologist to serve as the gold standard in supervised learning. The trained model was tasked with segmenting the test set for comparison with the manual segmentation results. The average Dice similarity coefficient in these comparisons was 84.7%.

**Conclusions:**

The proposed scheme for the automated segmentation and quantification of brain edema post-radiosurgery demonstrated excellent results, suggesting its applicability to the development of predictive models.

**Trial registration:**

Not applicable.

**Supplementary Information:**

The online version contains supplementary material available at 10.1186/s12880-025-01660-x.

## Introduction

Meningioma is the second most common primary tumor of the central nervous system after glioma. Its clinical presentation varies widely from quiescence to profound disability [1, 2]. Surgical resection is the standard of care for those with attributable symptoms or tumor growth [3]. Tumors proximal to eloquent areas or situated in surgically difficult areas are prone to distressing morbidity or even mortality. Thus, a balance must be struck between the advantages of surgical intervention and the corresponding risk.

Over the last 30 years, stereotactic radiosurgery (SRS) has emerged as an effective alternative treatment for lesions of the central nervous system [4]. Numerous studies have reported favorable results in using SRS to deal with meningioma [5–7]. In light of the radiological response and minimal adverse radiologic effect (ARE), the current consensus is to use SRS for tumors that are small, deep-seated, and/or asymptomatic [6].

ARE is a common phenomenon after SRS. The phenotype is best assessed using T2-weighted (T2w) magnetic resonance imaging (MRI) or fluid-attenuated inversion recovery (FLAIR) MRI, based on prominent features of peritumoral hyper-density indicative of edema. The clinical manifestation of brain edema ranges from asymptomatic to pronounced disability. Locoregional symptoms of brain edema depend on the involvement of functional areas comprising motor weakness, sensory disturbance, or language impairment. Generalized symptoms of edema include headache, conscious disturbance, seizure, or nausea. Treatments for ARE-related symptoms include a temporary course of steroids or bevacizumab, or surgical debulking in severe cases [8].

Peritumoral edema is clearly identifiable by hyper-dense areas in T2w MRI brain scans; however, physicians lack an objective tool by which to determine the volume of these areas. Volumetric analysis of these regions of interest has conventionally been performed by neuroradiologists; however, this process is lengthy, and the results are often non-reproducible. From a clinical perspective, a suspected correlation between edema volume and symptomatic manifestation (based on anecdotal findings) could potentially be used to predict long-term neurological outcomes [9]. Nonetheless, physicians require an early indication of ARE if they are to treat these difficult cases effectively.

The aim of this study was to automate the segmentation and quantification of post-radiosurgery brain edema using a deep learning-based model. Our findings represent a promising step toward enhanced edema segmentation accuracy and assessing the long-term effects of radiation therapy on surrounding brain tissue.

## Materials and methods

### Subjects

MRI data were collected from 21 patients at Taipei Veterans General Hospital. This included a total of 154 scans obtained at regular intervals (1 to 16 scans per patient). The average volume of cerebral edema after radiosurgery for meningioma was 15.61 ± 16.98 cm^3^, ranging from 0 to 139.66 cm^3^. Note that 24 scans presented edema with a volume of less than 2 cm^3^, which were excluded due to difficulties in delineation or other training-related reasons. After excluding those data from the training and validation datasets, the average volume was 18.24 ± 17.11 cm^3^, ranging from 2.01 to 139.66 cm^3^. The average patient age was 63.5 ± 9.1, ranging from 43 to 85 years. All scans were randomly divided into training, validation, and test sets. To ensure the independence of the three datasets, each patient was included in only one dataset. This prevented the occurrence of the same tracking scans appearing in different sets, thereby ensuring that the model was not tested using edema patterns on which it had previously been trained. Finally, we divided the dataset into five mutually exclusive subsets. In each iteration, four subsets were combined for training and validation, while the remaining subset was retained as a test set. This process was repeated five times, with each subset serving as the test set once. The final performance was averaged across the five test sets. The study was approved by the Institutional Review Board of Taipei Veterans General Hospital (2018-07-019 C).

### MRI protocol

Post-radiosurgery changes in perifocal edema, appearing as hyper-dense areas in magnetic resonance T2w images, are clearly differentiable from normal scans. We sought to increase the variety of data in order to enhance the robustness of the model to overfitting by importing images from several types of MRI scanners operating under various scanning parameters: repetition time = 2050-8854.7 ms, echo time = 82.3-140.8 ms, field of view = 70–100 mm, flip angle = 90-180^o^, number of averages = 1–4, and acquisition number = 0–4. The T2w images also varied in terms of dimensions and voxel size.

### Proposed algorithm

**Fig. 1:**
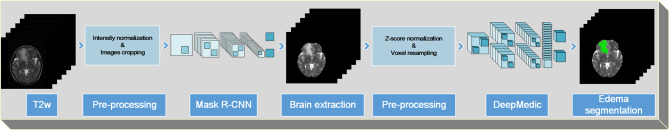
Flowchart of the proposed model for the segmentation of brain edema after radiosurgery for meningioma

To enhance the performance of the deep learning model, we employed transfer learning, which is more convenient, cost-effective, and efficient than developing a new model. As shown in Fig. 1, the generation of brain edema segmentations from T2w images was a 3-step process: (1) MRI pre-processing, (2) brain parenchyma extraction, and (3) segmentation of edema for quantification. Each step is detailed in the following sub-sections. The model was run on a personal computer equipped with an Intel CoreTM i7-10700 K CPU at 3.80 GHz and 16GB of RAM. The segmentation network was trained over a period of 18 h using an Nvidia RTX 3070Ti GPU with 8GB of RAM.

### MRI pre-processing

MRI pre-processing of T2w images was performed to improve computational efficiency and enhance the image analysis capabilities of the neural network to facilitate the extraction of as much lesion-related information as possible. Pre-processing involved z-score normalization, voxel size resampling, and image resizing.

T2w intensity normalization was meant to enhance the robustness and reliability of the results and accelerate convergence by reducing inter-rater bias [10]. All scans underwent voxel size resampling to 0.47 × 0.47 × 1.5 mm^3^ to facilitate segmentation at the voxel level. To build a deeper network of greater complexity, we increased the number of slices in the z-axis direction. In other words, we expanded the input volume size along the z-axis during pre-processing, by including additional adjacent slices in each sample to provide more contextual information for the 3D segmentation model.

This approach was meant to facilitate the capture of the spatial characteristics of brain edema across neighboring slices, which has been shown to improve segmentation performance in volumetric medical imaging tasks [11, 12]. We also performed image resizing to remove excess background information, potentially containing noise artifacts from the scanner.

### Data augmentation

In this study, data augmentation refers to the process of generating additional training samples by applying transformations to existing images, rather than obtaining new images from different slice locations.

We applied the following augmentation techniques to each T2-weighted image:

- Brightness Adjustment (Contrast Enhancement): Image intensity was randomly adjusted to simulate variations in scanning conditions [13].
- Elastic Deformation: Non-linear elastic transformations were applied to mimic subtle anatomical variations and scanner-induced distortions.

These augmentation techniques were applied independently to each image slice, resulting in additional versions of the same image with slight variations. Note that these operations did not afect the voxel information in any way that would alter anatomical structures. Instead, we introduced small variations to improve the robustness and generalizability of the segmentation model to unseen data.

The effectiveness of elastic transformation as a data augmentation method can be attributed to its simulation of natural variations that occur when medical images are originally generated. Variations in position, angle, and scanner parameters often result in slight stretching or other forms of distortion, such that the appearance of any medical image may vary under different screenings. Nonetheless, distortions of this sort should not influence the detection and identification of lesions. Numerous researchers have reported on the efficacy of elastic transformation in the modeling of variations for data augmentation [14].

As outlined [15], deformations were created by generating uniformly distributed random displacement fields Δx(x, y) = rand(-1,1) and Δy(x, y) = rand(-1,1). The expression rand(-1, 1) refers to a random number uniformly sampled from the range [-1, 1]. It is a dimensionless value and does not directly correspond to a physical displacement in millimeters. Rather, this random value is used in generating a displacement field for elastic deformation data augmentation.

The displacement field undergoes convolution with a Gaussian filter (regulated by elasticity coefficient σ), after which the final displacement is scaled by factor α. These parameters determine the physical extent of the deformation in voxel units. For instance, a displacement value of Δy = 1 represents a shift of 1 voxel in the y-direction, rather than a 1 mm displacement in physical space. The actual displacement in millimeters depends on the voxel size in the images, which was 0.47 mm × 0.47 mm × 1.5 mm after resampling.

### Brain parenchyma extraction

To enhance the efficiency and accuracy of the brain edema segmentation model, we employed the Mask R-CNN model to generate brain masks with parenchymal brain tissue as regions of interest for network modeling [16] (*Matterport*,* Inc*. (2018). Sunnyvale, CA. [Online]. Available: https://github.com/matterport).

Mask R-CNN is a pixel-level object detection and instance segmentation model, which won the Common Objects in Context (COCO) 2016 challenge. The model architecture is based on Fast/Fast R-CNN [17, 18] and a fully convolutional network [19]. This model is able to classify objects in pixels and simultaneously detect multiple types of objects for segmentation, with the results presented in the form of a semantic segmentation mask of very high accuracy. It is also highly efficient in terms of model training and inference during the brain mask extraction step. Mask R-CNN framework is publicly available at GitHub (https://github.com/matterport).

This study used a total of 4,049 T2w images for brain parenchyma extraction. This included 2,994 images in the training set, 710 in the validation set, and 345 in the test set. Statistical Parametric Mapping 12 (SPM12, Wellcome Trust Centre for Neuroimaging, University College London, https://www.fil.ion.ucl.ac.uk/spm/software/spm12/) [20] was used to generate brain mask labels, with missing parts filled in manually. The results that passed assessment by clinical physicians were adopted as the gold standard for subsequent analysis.

## Brain edema segmentation

DeepMedic is a multi-scale 3D deep convolutional neural network with 3D fully connected conditional random fields designed for the segmentation of 3D medical images (*DeepMedic*. (2019). Oxford UK. [Online]. Available: https://github.com/deepmedic). When applied to MRI scans, this model has proven highly effective in segmenting lesions associated with traumatic brain injury, ischemic stroke, and meningioma [21]. This model was also the winner of the Brain Tumor Image Segmentation (BRATS) 2015 and Ischemic Stroke Lesion Segmentation (ISLES) 2015 benchmarks. The DeepMedic architecture is based on a multi-scale deep convolutional neural network and fully connected conditional random fields, which are highly effective in removing false positives during the segmentation stage [22, 23]. The input employs two parallel convolution channels to extract lesion-related image features, capturing both local details and large-scale contour information across multiple scales. Class imbalances can be mitigated by dense training in the fully convolutional network. The DeepMedic framework is publicly available at GitHub (https://github.com/deepmedic).

In the current study, the DeepMedic model was used as the primary edema segmentation tool. Transfer learning was used to adjust the weights, and training hyperparameters were used in the segmentation and quantification of edema in T2w images. The ability of DeepMedic to process 3D MRI data allowed the incorporation of image features along the z-axis for use in assessing the long-term effects of radiation therapy on brain tissue surrounding meningiomas.

## Performance evaluation

This study used three common evaluation indices to assess the accuracy of the segmentation model based on its ability to differentiate between automated segmentation results and the ground truth (manual delineation by radiologists). These indices included the Dice similarity coefficient (DSC), precision, and recall. This analysis was based on the four elements of a confusion matrix: true positive (TP), false positive (FP), true negative (TN), and false negative (FN).

The definitions used in this study were as follows:

- True Positive (TP): A voxel labeled as edema by both the model and the radiologist.
- False Positive (FP): A voxel labeled as edema by the model but not by the radiologist.
- True Negative (TN): A voxel labeled as non-edema by both the model and the radiologist.
- False Negative (FN): A voxel labeled as non-edema by the model but identified as edema by the radiologist.

A voxel was considered correctly segmented if its label (edema or non-edema) matched the ground truth determined by a radiologist. DSC measures the degree of similarity between two samples in terms of shape, area, and position. Precision assesses the probability that a positive prediction is actually true, emphasizing the accuracy of predicted positive outcomes. Recall assesses the probability that an actual positive case is correctly identified by the model, focusing on the accuracy of true positive predictions.

Taken together, these indices can detect whether the predictions of a segmentation model correspond to reality.

## Results

### Demographics

A total of 21 patients were recruited in the study. This sample included a preponderance of females (*n* = 17, 81%). The average age at the time of clinical presentation was 63 years old, ranging from 43 to 81 years. A notable portion of the patients in this series were incidentally diagnosed with meningioma without any symptoms (*n* = 8, 38%). Other attributable ailments included headache (*n* = 6, 29%), ocular phenomena (*n* = 3, 14%), hearing problems (*n* = 3, 14%), and seizure (*n* = 1, 5%). All cases involved isolated tumors, such that this population yielded a total of 21 meningiomas, 67% of which were deep-seated in the brain parenchyma (skull base, *n* = 10; cerebellopontine angle, *n* = 2; tentorium, *n* = 1; intra-ventricle, *n* = 1). The mean tumor size was 7.03 cm^3^ at baseline, ranging from 1.60 to 15.92 cm^3^. Table 1 summarizes the clinical presentations and imaging phenotypes.

**Table 1:** Characteristics of the 21 meningioma patients in this study

Factor	Value
Male	4
Median age in years (range)	63 (43–81)
Initial presentation	
Headache	6
Seizure	1
Blurred vision	2
Diplopia	1
Hearing impairment	2
Tinnitus	1
Incidental finding	8
Location	
Falx	3
Para-sagittal	2
Convexity	2
Tentorium	1
Skull base	10
Cerebellopontine angle	2
Intra-ventricle	1
Tumor volume in cm^3^ (range)	7.03 (1.60-15.92)

MRI scans were used to perform volumetric analysis of the meningioma. Note that meningioma can generally be enhanced by contrast media. The target volume of SRS can be delineated clearly using post-contrast T1-weighted MRI scans. This can then be used to guide dose delivery planning. In the series, a marginal dose fell within a relatively narrow spectrum of 11.5 to 13 Gy. The average time that elapsed between the last SRS session and maximal brain edema was 13 months, spanning 3.3 to 64 months. The maximal calculated edema volume ranged from 1.40 to 139.66 cm^3^. The SRS treatment parameters and outcomes are listed in Supplementary Table 1.

### Automated brain parenchyma extraction

The performance of the Mask R-CNN model in brain parenchyma extraction was evaluated using five-fold cross-validation. The model achieved consistently high performance, with an average Dice similarity coefficient (DSC) of 94.98%, recall of 92.51%, and precision of 97.89% across all folds. Detailed results for each fold are presented in Supplementary Table 2.

Correct extraction of brain parenchyma was defined voxel-wise. A voxel was considered correctly extracted if it was classified as brain parenchyma by the model and matched the corresponding voxel in the ground truth mask, which was manually labeled by a radiologist. Supplementary Fig. 1 presents a demonstration of brain parenchyma extraction using the Mask R-CNN.

### Automated edema segmentation

The performance of the DeepMedic model in edema segmentation was also evaluated using five-fold cross-validation. The average Dice similarity coefficient (DSC) across all folds was 80.51%, with a recall of 75.20% and precision of 88.46%. Detailed performance metrics for each fold are provided in Table 2.

**Table 2:** Five-fold cross-validation results of the proposed model in the segmentation of brain edema

Fold	Set	Scans	EV (ml)	DSC (%)	Recall (%)	Precision (%)
1	Validation	25	16.9 ± 20.8	83.59-	76.31	92.88
	Test	18	18.1 ± 9.4	81.19	74.01	90.24
2	Validation	25	17.7 ± 12.1	78.81	74.45	89.15
	Test	17	14.4 ± 8.1	79.21	73.45	87.77
3	Validation	26	17.7 ± 26.4	75.37	68.19	89.65
	Test	17	18.2 ± 13.7	73.82	66.31	88.48
4	Validation	22	17.4 ± 21.4	86.88	84,92	86.94
	Test	14	18.4 ± 11.6	84.74	83.96	85.49
5	Validation	24	16.8 ± 20.4	83.84	80.44	91.05
	Test	12	16.0 ± 7.9	83.58	78.26	90.31
Mean ± STD	Validation			81.23 ± 5.13	74.85 ± 5.10	89.93 ± 2.21
	Test			80.51 ± 4.31	75.20 ± 6.51	88.46 ± 1.99

STD: Standard deviation

**Fig. 2:**
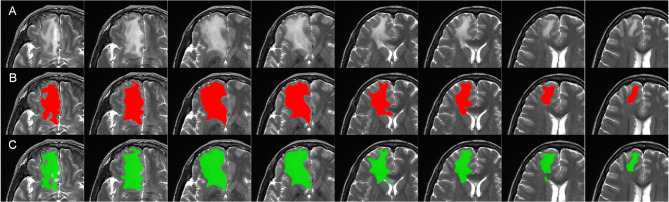
Brain edema segmentation results: (**a**) T2w image, (**b**) Ground truth segmentation (shown in red), and (**c**) Automated edema segmentation (shown in green)

Following cross-validation, the final model used in this study was selected based on the fold that achieved the highest DSC on the test set. This model served as the basis for future applications and inference. Figure 2 presents a demonstration of edema segmentation by DeepMedic.

### Edema progression

**Fig. 3:**
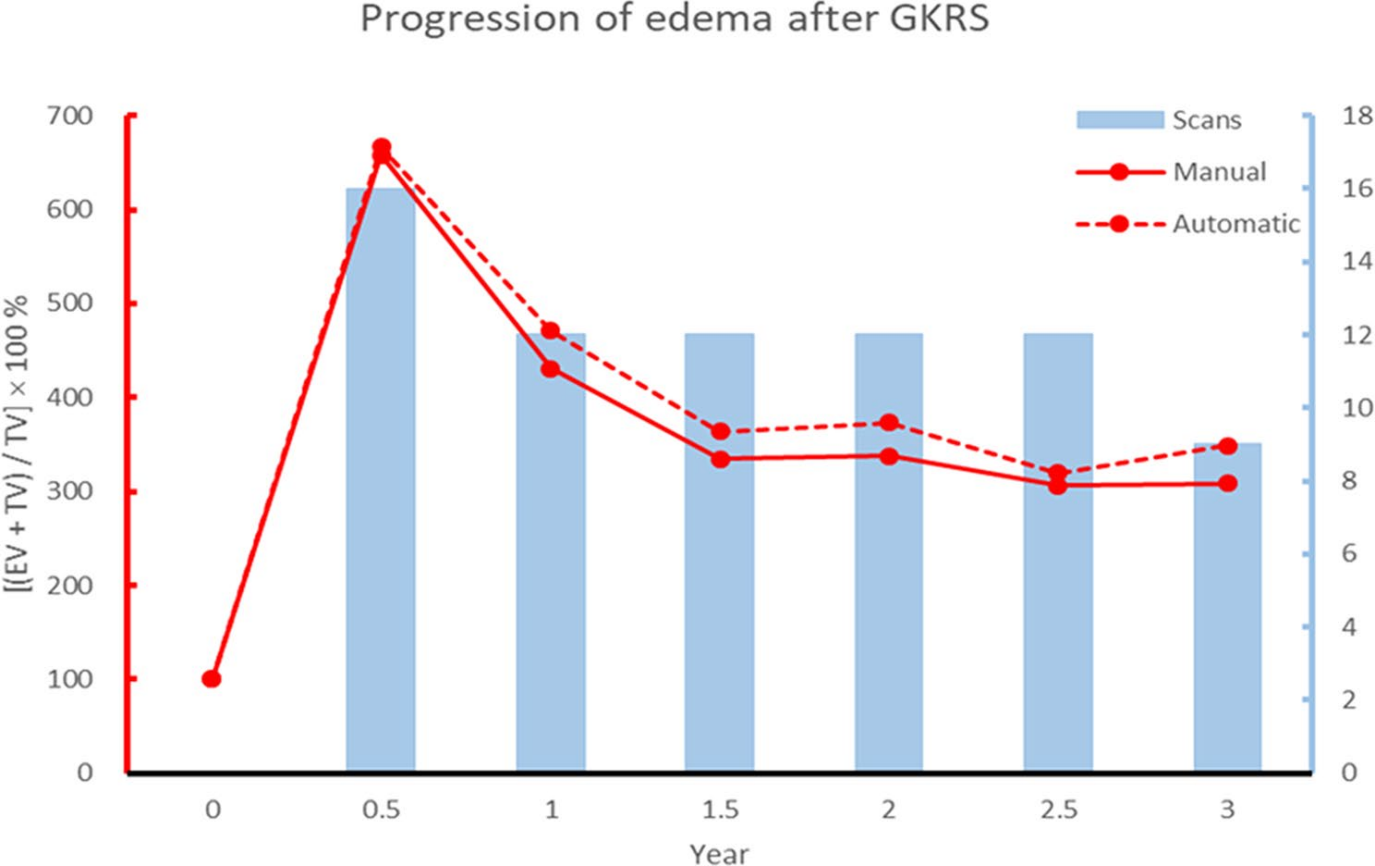
Post-GKRS progression of edema (manual versus automated assessment) and the number of scans used for statistical analysis. $\:{EV}_{stand.}=\:\frac{\left[\left(EV+TV\right)\right]}{TV}\times\:100$%, where EV refers to edema volume, and TV refers to tumor volume

All scanning data were tracked at intervals of six months over a period of three years. After normalizing the volume of brain edema and meningiomas, a progression chart (Fig. 3) was created to reveal changes in brain edema following GKRS. The trend in edema volumes quantified via automated segmentation closely matched the manual markings. The observation that edema volume peaked after GKRS and plateaued after 18 or 24 months is an interesting discovery and in good agreement with our predictions. This could be valuable in formulating a model for the prediction of long-term changes in perifocal brain edema after radiosurgery. It should also enable comparisons of edema severity among patients.

## Discussion

Volumetric analysis of peritumoral edema based on serial imaging provides a wealth of objective data for tracking intracranial conditions. Yen et al. proposed a semi-quantitative grading system for the stratification of post-SRS ARE: Grade I (mild imaging changes without mass effect), Grade II (effacement of the sulci or compression of the ventricles), and Grade III (shift in the midline of the brain) [8].

Dynamic changes in peritumoral edema volume could serve as an indicator of clinical trajectory, guiding clinical decision-making regarding the choice of intervention (e.g., steroid or surgical decompression). There is a clear need for methods to identify patients at risk of post-SRS ARE. T2w MRI scans provide the clearest indications of ARE, as evidenced by high-intensity signals denoting edematous change. For decades, edema volume has been calculated manually by radiologic specialists; however, this process is tedious, time-consuming, and subject to variability, particularly in cases of poor lesion visualization.

In the current study, we developed a deep learning algorithm to automate the segmentation and quantification of brain edema following Gamma Knife radiosurgery (GKRS) for meningioma.

In experiments, the proposed deep learning model achieved high segmentation accuracy, comparable to manual delineation by radiologists. Nonetheless, it was observed that segmentation failures were more common in cases involving small edema volumes (< 2 cm³) or edema regions with irregular and indistinct margins. These patterns suggest that the model may struggle with subtle or ill-defined edema boundaries, a known challenge in medical image segmentation.

Successful predictions were more likely in cases with larger, well-demarcated edema, which provided clearer intensity contrast in T2-weighted images. Consistent image quality across longitudinal scans also appeared to enhance model performance, reducing variability in the segmentation process. Longitudinal analysis revealed that edema volume typically peaks at roughly 13 months after GKRS and stabilizes after 24 months.

Accurate volumetric assessment is critical, as cases with disproportionately large edema relative to tumor volume may require closer monitoring and early intervention. In some cases, the post-radiosurgery edema volume reached 600% of the tumor volume (see Fig. 3). These cases were associated with significant clinical symptoms, such as motor weakness, headache, or cognitive disturbances, necessitating medical interventions such as corticosteroid therapy. This aligns with prior observations and suggests that automated volumetric assessment could enhance clinical monitoring and decision-making.

Physicians commonly encounter patients who are susceptible to progressive post-SRS ARE, which often requires aggressive intervention [24]. In a retrospective review of patients who underwent SRS for meningioma, Sheehan et al. reported that the interval to peak tumor volume could be used to differentiate between cases of transient brain edema (peaking at 18 months) and cases of progressive edema (peaking at 36 months) [25]. Those findings highlight the importance of longitudinal clinical and radiographic follow-up after SRS treatment.

The proposed dep-learning model provides reliable segmentation results in a timely manner for informed clinical decision-making. Although this study focused exclusively on brain edema segmentation, the underlying automated segmentation technique could potentially be extended to other applications, such as meningiomas—the most common primary brain tumor—or other tumor-associated edema cases, thereby increasing its clinical utility.

Despite promising results, this study was subject to various limitations. First, the small sample size and the predominance of skull base tumors may limit generalizability. Second, the model was trained using a dataset from a single medical center, which may affect performance when applied to data from other institutions. Future work will focus on expanding the dataset, validating the model on multi-center data, and integrating additional imaging modalities to improve robustness.

## Conclusions

This study trained two different deep convolution neural networks with the aim of separating the segmentation process into distinct functions. Our experiment results demonstrate the feasibility, reliability, and effectiveness of this approach. Automating the process of skull and scalp stripping is a crucial step in interpreting medical images. Segmenting different lesions in MRI scans can facilitate the model-building process, and accurate segmentation of brain edema is critical to quantifying the long-term effects of radiation therapy on the tissue surrounding meningiomas. The fully automated segmentation process proposed in this study provides accuracy on par with that of experienced professionals.

## Electronic supplementary material

Below is the link to the electronic supplementary material.

Supplementary Material 1

Supplementary Material 2

Supplementary Material 3

Supplementary Material 4

## Data Availability

The datasets used and/or analyzed during the current study are available from the corresponding author on reasonable request.

## Abbreviations

ARE: Adverse radiologic effect
DSC: Dice similarity coefficient
FLAIR: Fluid-attenuated inversion recovery
GKRS: Gamma Knife radiosurgery
Mask R-CNN: Mask Region-based Convolutional Neural Network
MRI: Magnetic resonance imaging
SRS: Stereotactic radiosurgery
T2w: T2-weighted
