# Supplementary material for: Deep learning for automated segmentation of brain edema in meningioma after radiosurgery
Source: BMC Med Imaging. 2025 Apr 22;25:130. doi: 10.1186/s12880-025-01660-x (PMC12016358; doi:10.1186/s12880-025-01660-x)
Supplement: Supplementary file 1 — Supplementary Material 1 [file 12880_2025_1660_MOESM1_ESM.docx]

Supplementary Table 1. Treatment parameters and imaging results of 21 patients treated using SRS for brain meningioma.

| **Case** | **Meningioma location** | **Radiation treatment volume (cm^3^)** | **Margin dose (Gy)** | **Max dose (Gy)** | **Maximal edema volume (cm^3^)** | **Interval between SRS and maximal edema (months)** |
| --- | --- | --- | --- | --- | --- | --- |
| 1 | Frontal base | 3.60 | 12 | 20.5 | 21.22 | 64.37 |
| 2 | Frontal base | 4.00 | 12 | 22 | 33.53 | 3.03 |
| 3 | Parietal falx | 10.20 | 12 | 21 | 25.97 | 13.70 |
| 4 | Frontal pole | 4.10 | 13 | 21.9 | 11.80 | 17.57 |
| 5 | Parietal convexity | 6.10 | 12.5 | 20.9 | 17.85 | 6.70 |
| 6 | Temporal pole | 2.63 | 12 | 20.8 | 12.13 | 7.40 |
| 7 | Frontal base | 6.56 | 12 | 23 | 27.12 | 12.93 |
| 8 | Mesial temporal region | 8.60 | 12 | 20.8 | 58.25 | 18.33 |
| 9 | Occipital falx | 4.50 | 13 | 21.3 | 58.41 | 5.63 |
| 10 | Mesial temporal region | 6.50 | 12 | 21.6 | 41.51 | 11.87 |
| 11 | Frontal base | 9.20 | 12 | 21.3 | 100.19 | 5.87 |
| 12 | Petroclival region | 3.96 | 12 | 23.3 | 6.11 | 17.00 |
| 13 | Parietal convexity | 5.30 | 12 | 21.3 | 28.61 | 14.73 |
| 14 | Cerebellopontine angle | 8.50 | 12.5 | 20.9 | 25.38 | 4.50 |
| 15 | Frontal base | 1.60 | 12 | 20.3 | 5.34 | 8.60 |
| 16 | Frontal falx | 7.30 | 12 | 20.1 | 139.66 | 5.73 |
| 17 | Occipital tentorium | 7.01 | 12 | 24.1 | 25.08 | 8.03 |
| 18 | Frontal base | 5.92 | 12 | 21.3 | 20.24 | 7.37 |
| 19 | Cerebellopontine angle | 9.63 | 12 | 22.6 | 1.40 | 12.20 |
| 20 | Occipital ventricle | 15.92 | 12 | 23 | 48.08 | 6.93 |
| 21 | Parietal para-sagittal | 16.50 | 11.5 | 21.5 | 3.58 | 20.20 |
