# Supplementary material for: Deep learning for automated segmentation of brain edema in meningioma after radiosurgery
Source: BMC Med Imaging. 2025 Apr 22;25:130. doi: 10.1186/s12880-025-01660-x (PMC12016358; doi:10.1186/s12880-025-01660-x)
Supplement: Supplementary file 2 — Supplementary Material 2 [file 12880_2025_1660_MOESM2_ESM.docx]

Supplementary Table 2. Five-fold cross-validation results of the proposed model in the extraction of brain parenchyma.

| **Fold** | **Subset** | **Slices** | **DSC (%)** | **Recall (%)** | **Precision (%)** |
| --- | --- | --- | --- | --- | --- |
| **1** | Validation | 845 | 96.12 | 94.66 | 98.77 |
|  | Test | 671 | 95.15 | 92.62 | 98.18 |
| **2** | Validation | 689 | 96.59 | 93.89 | 98.21 |
|  | Test | 565 | 95.07 | 92.50 | 98.04 |
| **3** | Validation | 706 | 94.92 | 93.31 | 97.46 |
|  | Test | 522 | 93.27 | 90.92 | 96.02 |
| **4** | Validation | 602 | 96.22 | 93.59 | 98.41 |
|  | Test | 513 | 94.68 | 92.15 | 97.92 |
| **5** | Validation | 710 | 97.17 | 95.77 | 98.79 |
|  | Test | 345 | 96.72 | 94.34 | 99.27 |
| **Mean ± STD** | Validation |  | 96.20 ± 0.83 | 94.24 ± 0.99 | 98.33 ± 0.54 |
|  | Test |  | 94.98 ± 1.23 | 92.51 ± 1.23 | 97.89 ± 1.17 |

STD: Standard deviation.
