# Supplementary figures and images for: Deep learning for automated segmentation of brain edema in meningioma after radiosurgery
Source: BMC Med Imaging. 2025 Apr 22;25:130. doi: 10.1186/s12880-025-01660-x (PMC12016358; doi:10.1186/s12880-025-01660-x)

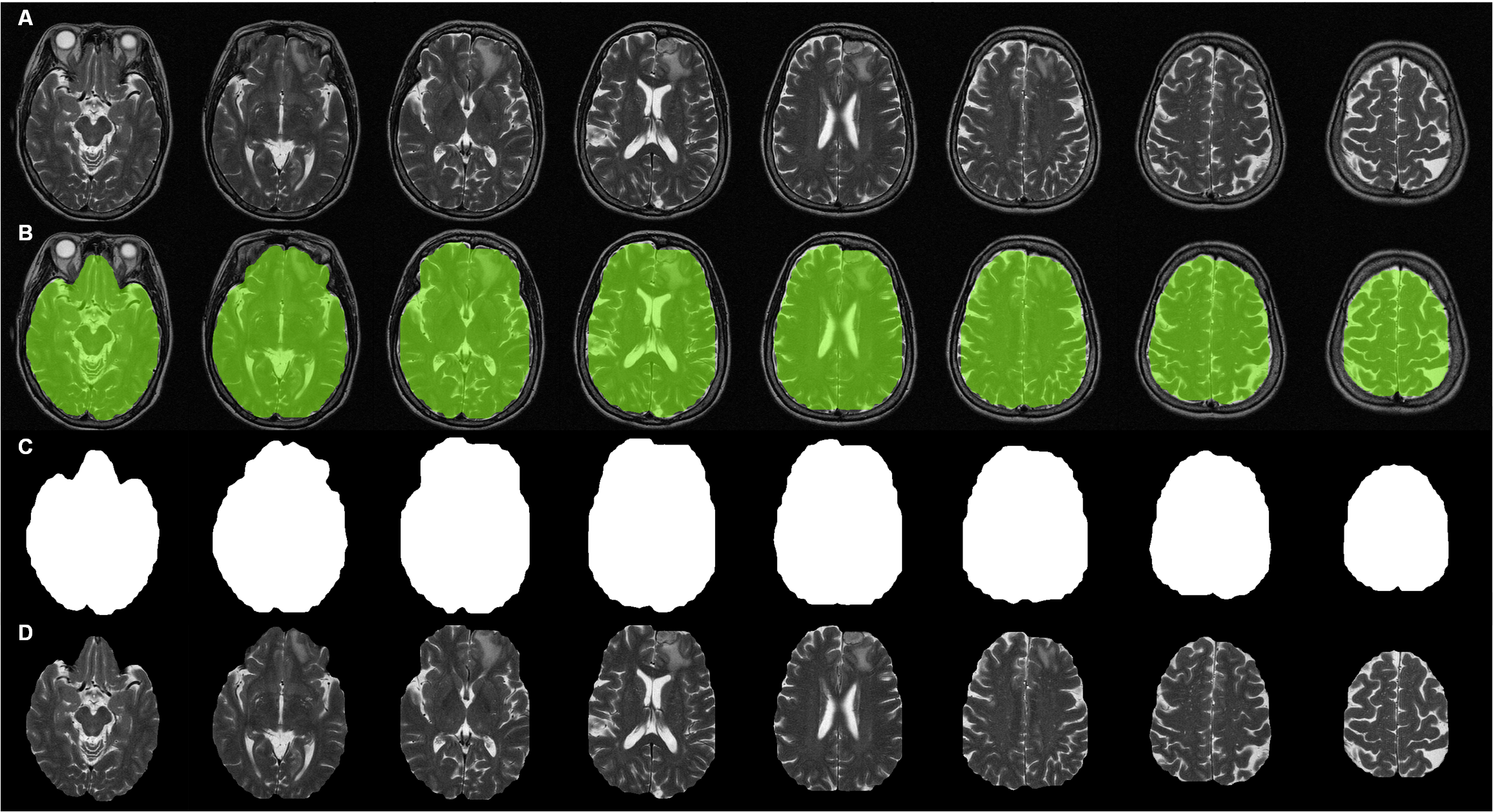

Supplement: Supplementary file 3 — Supplementary Material 3 [file 12880_2025_1660_MOESM3_ESM.tif]
