# Supplementary material for: Deep learning for automated segmentation of brain edema in meningioma after radiosurgery
Source: BMC Med Imaging. 2025 Apr 22;25:130. doi: 10.1186/s12880-025-01660-x (PMC12016358; doi:10.1186/s12880-025-01660-x)
Supplement: Supplementary file 4 — Supplementary Material 4 [file 12880_2025_1660_MOESM4_ESM.docx]

**Supplementary figure legend**

**Supplementary Figure 1** Automated extraction of brain parenchyma: (a) Raw T2w images, (b) Manual segmentation masks, (c) Mask R-CNN automated masks, and (d) Automated skull and scalp stripping.
